# Targeting Ikaros and Aiolos: Next‐Generation Cereblon E3 Ligase Modulators in MM

**DOI:** 10.1111/ejh.70134

**Published:** 2026-02-06

**Authors:** Maria Eugenia Alvaro, Enrica Antonia Martino, Santino Caserta, Mamdouh Skafi, Antonella Bruzzese, Nicola Amodio, Eugenio Lucia, Virginia Olivito, Caterina Labanca, Francesco Mendicino, Ernesto Vigna, Fortunato Morabito, Massimo Gentile

**Affiliations:** ^1^ Department of Onco‐Hematology AO of Cosenza, Hematology Unit Cosenza Italy; ^2^ Emergency and Internal Medicine Department Saint Joseph Hospital East Jerusalem Palestine; ^3^ Department of Experimental and Clinical Medicine University of Catanzaro Catanzaro Italy; ^4^ AIL Sezione di Cosenza Cosenza Italy; ^5^ Department of Pharmacy Health and Nutritional Science, University of Calabria Rende Italy

**Keywords:** Cereblon E3 ligase modulators (CELMoDs), Iberdomide, IMiD resistance/immunomodulation, Mezigdomide, multiple myeloma

## Abstract

Multiple myeloma (MM) remains an incurable plasma cell malignancy characterized by recurrent relapses and eventual refractoriness to standard agents, including proteasome inhibitors (PIs), immunomodulatory drugs (IMiDs), and anti‐CD38 monoclonal antibodies. With frontline therapeutic strategies increasingly employing quadruplet induction regimens and prolonged lenalidomide maintenance, resistance to traditional IMiDs has become more prevalent, creating an urgent need for next‐generation cereblon E3 ligase modulators (CELMoDs) capable of overcoming IMiD refractoriness and enhancing the immunologic microenvironment. Iberdomide (CC‐220) and mezigdomide (CC‐92480) are rationally engineered CELMoDs designed to achieve deeper degradation of Ikaros (IKZF1) and Aiolos (IKZF3), restore cereblon‐mediated activity, and potentiate immune effector responses. This review explores the core biological features of these agents, detailing their mechanisms of action, preclinical and clinical activity, as well as safety profile. We examine how their pharmacodynamic properties differ from classical IMiDs, their relevance in triple‐class and penta‐refractory MM, and their integration into emerging combination strategies with monoclonal antibodies and T‐cell–redirecting immunotherapies. Special emphasis is placed on ongoing and future trials that may refine their therapeutic positioning, alongside a critical appraisal of the limitations and future directions of this rapidly advancing drug class.

## Graphical abstract

The graphical abstract illustrates the mechanism of action of cereblon E3 ligase modulators (CELMoDs) in multiple myeloma, highlighting cereblon‐mediated degradation of IKZF1 and IKZF3. Compared with classical immunomodulatory drugs (IMiDs), CELMoDs induce deeper IKZF1/3 depletion, leading to more pronounced suppression of the IRF4–MYC survival axis and enhanced tumor‐intrinsic cytotoxicity. The figure also highlights the concurrent activation of innate and adaptive antitumor immune responses.

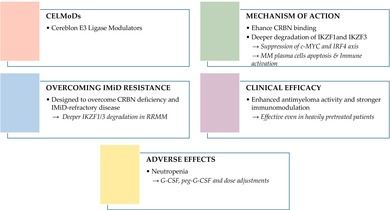

## 1 Introduction

Multiple myeloma (MM) is a clonal malignancy of plasma cells that accumulates in the bone marrow, leading to progressive immune dysfunction, end‐organ damage, and a complex interplay between the malignant clone and its microenvironment. It accounts for ~1% of all cancers and nearly 10% of hematologic malignancies, with a median age at diagnosis of around 70 years [1].

Although major therapeutic advances over recent decades have substantially improved survival, MM remains characterized by repeated cycles of remission and relapse. Its clinical course is intrinsically heterogeneous, driven by genetic lesions, epigenetic reprogramming, and microenvironmental interactions that shape disease behaviour and treatment response [2].

MM typically arises from an asymptomatic premalignant stage known as monoclonal gammopathy of undetermined significance (MGUS), which progresses into smouldering multiple myeloma and eventually symptomatic MM. Each transition corresponds to increased genomic complexity and profound remodelling in the bone marrow niche [3].

Malignant plasma cells promote immune escape by impairing T‐cell activation, downregulating antigen presentation pathways, expanding regulatory T cells (Tregs), recruiting myeloid‐derived suppressor cells (MDSCs), and reshaping macrophage polarization. These immunosuppressive mechanisms are central to both disease persistence and treatment resistance [4].

MM disproportionately affects older adults, with a median age at diagnosis of ~69–70 years. Age‐related comorbidities and immune decline frequently complicate therapeutic decisions and limit tolerance to intensive regimens. Despite markedly improved outcomes in younger and fitter patients, the disease remains essentially incurable, and cumulative treatment burden, frailty, and immune dysfunction significantly contribute to morbidity and mortality [5, 6, 7].

Historically, treatment relied on cytotoxic chemotherapy; however, current paradigms integrate proteasome inhibitors, IMiDs, CD38‐ and SLAMF7‐directed monoclonal antibodies, steroids, and, more recently, XPO‐1 inhibitors, conjugate antibodies, immune effector cell therapies such as CAR‐T cells and bispecific T‐cell engagers. IMiDs—thalidomide, lenalidomide, and pomalidomide—have been particularly transformative, providing durable responses through immune activation and microenvironment modulation [8, 9, 10, 11, 12, 13, 14, 15, 16, 17, 18]. A frontline therapy increasingly incorporates lenalidomide‐based quadruplets and extended maintenance strategies; IMiD resistance has emerged as an early and more prominent clinical challenge. Many patients who fail lenalidomide first relapse rapidly develop triple‐class refractoriness, often within only a few treatment lines [19]. This therapeutic bottleneck underscores the need for agents capable of restoring cereblon‐dependent degradation pathways and reactivating downstream immune and transcriptional networks [20, 21, 22, 23].

**FIGURE 1:**
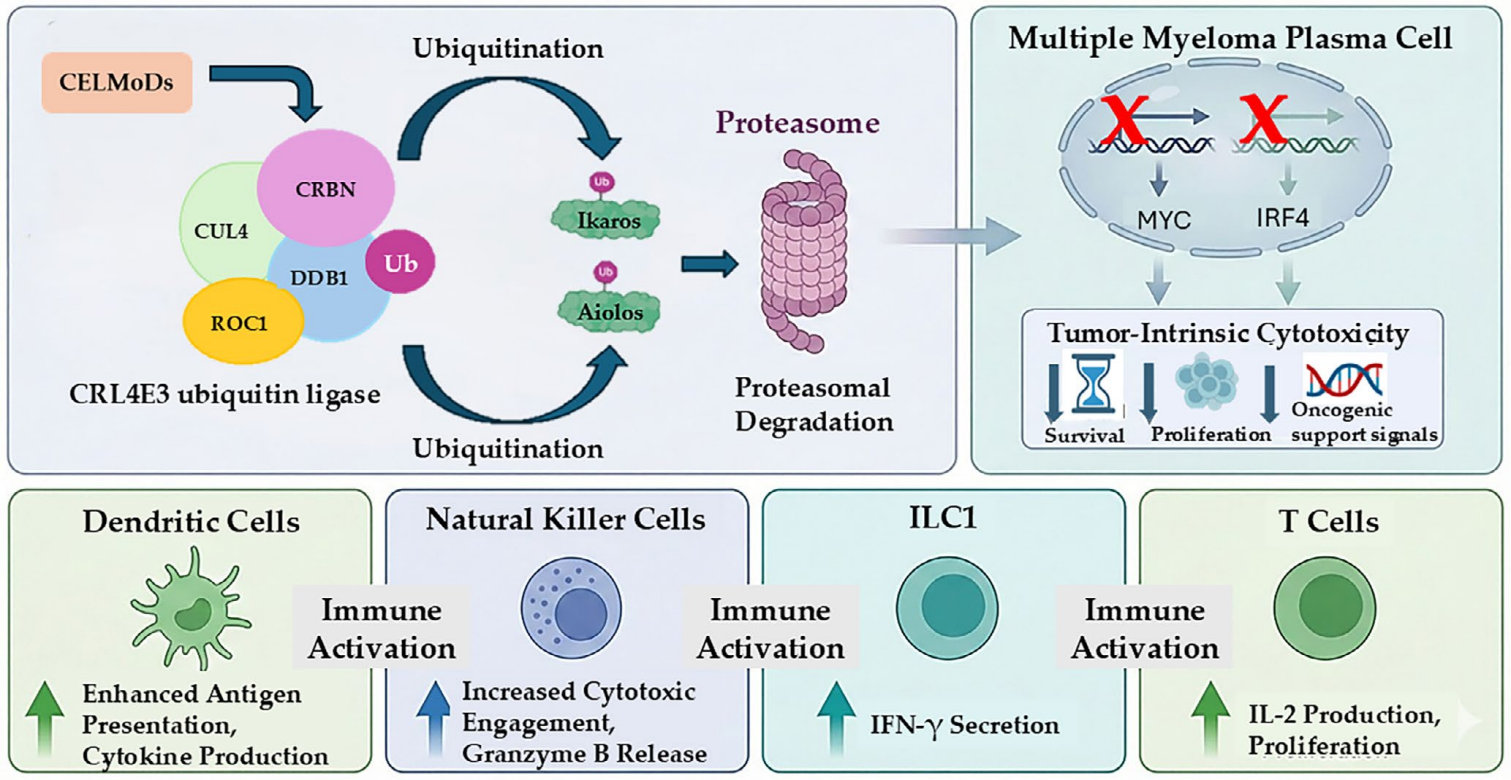
Degradation of IKZF1 and IKZF3: Impact on multiple myeloma. Cereblon E3 ligase modulators (CELMoDs) promote cereblon (CRBN)–mediated ubiquitination and proteasomal degradation of IKZF1 (Ikaros) and IKZF3 (Aiolos), resulting in suppression of the IRF4–MYC survival axis in multiple myeloma (MM) plasma cells and induction of tumor‐intrinsic cytotoxicity. Concurrently, IKZF1/3 depletion enhances antitumor immune responses by activating dendritic cells, natural killer (NK) cells, innate lymphoid cells type 1 (ILC1), and T cells, thereby contributing to the dual direct and immunomodulatory activity of CELMoDs. CELMoDs, cereblon E3 ligase modulators; CRBN, cereblon; IKZF, Ikaros zinc finger; ILC1, innate lymphoid cells type 1; NK, natural killer.

CELMoDs such as iberdomide and mezigdomide were developed to overcome these limitations. Engineered to bind cereblon with higher affinity, they promote more efficient degradation of IKZF1 (Ikaros) and IKZF3 (Aiolos), resulting in greater suppression of IRF4 and c‐MYC—central transcriptional regulators aberrantly co‐opted in MM to sustain malignant plasma cell survival. Unlike their tumor‐suppressive role in other hematologic contexts, IKZF1 and IKZF3 are highly expressed and functionally required in myeloma cells, where they maintain oncogenic transcriptional programs centered on the IRF4–c‐MYC axis. IRF4 and c‐MYC form a tightly interconnected, self‐reinforcing regulatory loop that drives proliferation, metabolic fitness, bone marrow retention, and resistance to apoptosis. Through enhanced cereblon engagement, CELMoDs induce deeper and more sustained depletion of Ikaros and Aiolos, leading to marked downregulation of IRF4 and c‐MYC, collapse of pro‐survival transcriptional programs, and induction of myeloma cell apoptosis. Concurrently, IKZF degradation reshapes the tumor–immune interface by enhancing interferon signaling, increasing susceptibility to immune effector mechanisms, and amplifying T‐ and NK‐cell–mediated antitumor activity [24]. Therefore, beyond direct cytotoxicity, CELMoDs enhance immune activation, boost T‐cell effector function, and strengthen NK‐cell–mediated antibody‐dependent cellular cytotoxicity (ADCC), making them particularly well‐suited partners for monoclonal antibodies, bispecific immunotherapies, and post–CAR‐T strategies (Figure 1) [25].

## 2 CELMoDs: Mechanism of Action and Pharmacology

The central mechanism of action of both IMiDs and CELMoDs is based on modulation of cereblon (CRBN), the substrate receptor within the CUL4‐RBX1‐DDB1 E3 ubiquitin ligase complex. Thalidomide analogues function as “molecular glue degraders,” creating new protein–protein interfaces that enable the recruitment of transcription factor neosubstrates. The discovery that IKZF1 (Ikaros) and IKZF3 (Aiolos) are primary cereblon‐dependent degradation targets of IMiDs fundamentally transformed the understanding of their antimyeloma activity [26].

CELMoDs were engineered to enhance ligand‐CRBN interactions through several structural innovations. While lenalidomide and pomalidomide bind CRBN primarily through a glutarimide ring, iberdomide and mezigdomide incorporate additional chemical extensions that enlarge the interaction surfaces and engage residues beyond the classical tri‐tryptophan pocket. These modifications promote a more stable CRBN–ligand conformation and induce an allosteric shift toward a fully closed, active CRBN state, optimizing the interface required for neosubstrate recruitment and ubiquitination [27].

This improved CRBN engagement achieved by CELMoDs results in significantly deeper and faster degradation of IKZF1 and IKZF3. These transcription factors regulate the expression of IRF4 and MYC, which together sustain proliferation, inhibit apoptosis, and maintain plasma cell differentiation. Degradation of IKZF1/3 dismantles oncogenic enhancer networks and disrupts the IRF4–MYC feedback loop, leading to transcriptional collapse and potent induction of apoptosis in malignant plasma cells [28].

**FIGURE 2:**
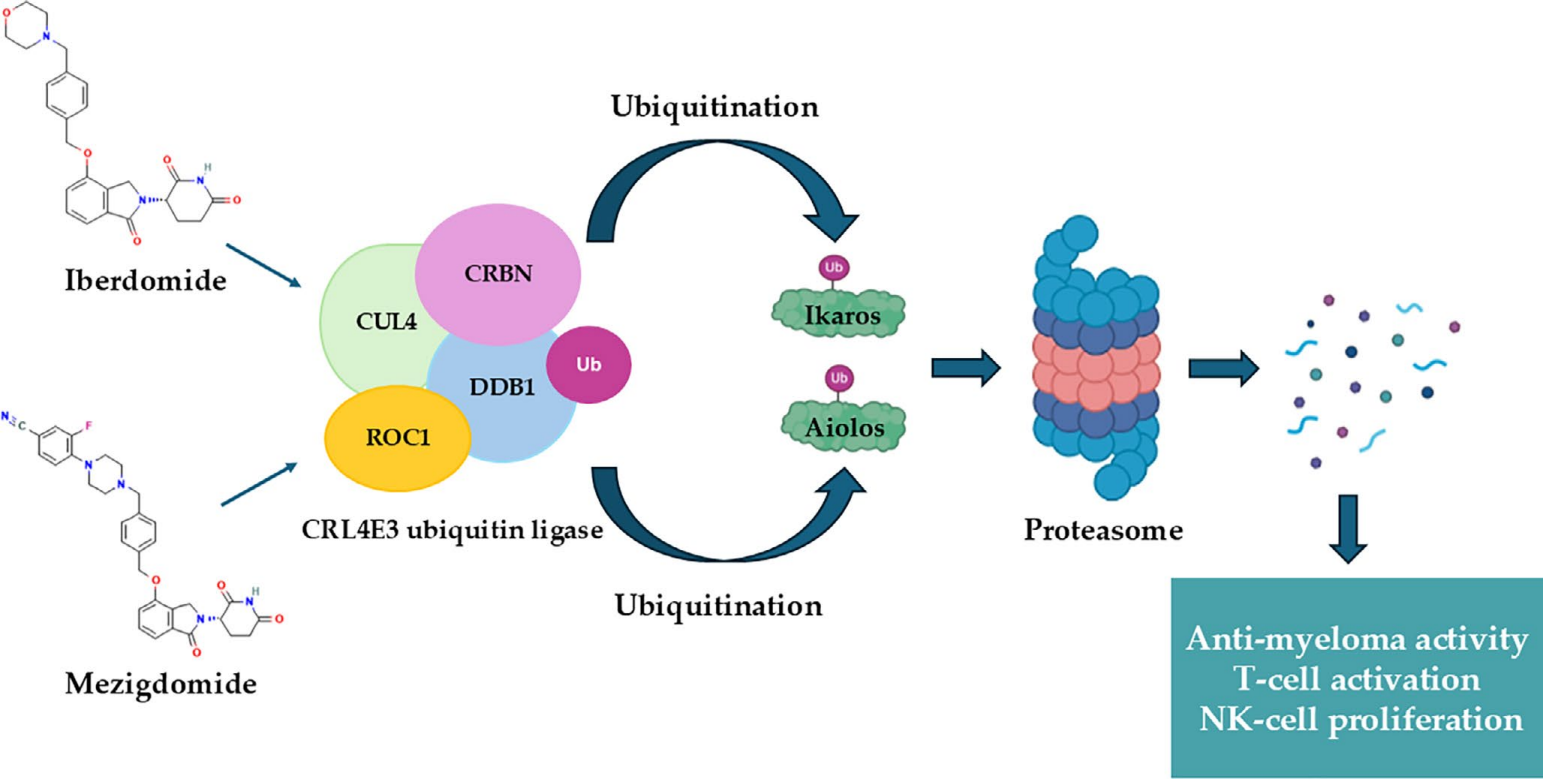
Mechanism of action of mezigdomide and iberdomide. Iberdomide and mezigdomide bind to cereblon within the CRL4 E3 ubiquitin ligase complex, enhancing recruitment and ubiquitination of the transcription factors Ikaros (IKZF1) and Aiolos (IKZF3). Polyubiquitinated substrates are subsequently degraded by the proteasome, resulting in suppression of IRF4/MYC‐dependent survival pathways and promoting downstream immunomodulatory effects, including augmented T‐cell activation, NK‐cell expansion, and anti‐myeloma activity. CRBN, cereblon; CUL4, cullin 4; DDB1, DNA damage‐binding protein; NK, natural killer; ROC1, regulator of cullins 1; Ub Ubiquitine.

The immunomodulatory properties of CELMoDs arise from the same molecular mechanism. IKZF1 and IKZF3 degradation reprograms T‐ and NK‐cell function, increases IL‐2 production, enhances T‐cell proliferation, and boosts cytotoxic effector responses. Iberdomide and mezigdomide induce more pronounced immune activation than lenalidomide or pomalidomide, supporting strong synergy with monoclonal antibodies, bispecific T‐cell engagers, and other immunotherapies (Figure 2) [29].

The potency of mezigdomide is particularly noteworthy. Its optimized structural design confers even higher affinity for cereblon than iberdomide, enabling more profound and consistent depletion of IKZF1/3. This depth of neosubstrate degradation explains Mezigdomide's efficacy in heavily pretreated, multidrug‐refractory MM, where traditional IMiDs and other agents have limited activity [30].

## 3 Preclinical Evidence

Preclinical investigations provide a strong foundation supporting clinical development of iberdomide and mezigdomide. Iberdomide has demonstrated potent and consistent depletion of IKZF1 and IKZF3 in both IMiD‐sensitive and IMiD‐resistant MM cell lines. Its ability to degrade these transcription factors at lower concentrations and with more rapid kinetics than lenalidomide or pomalidomide leads to more pronounced downregulation of IRF4 and MYC, thereby decreasing cell viability [31].

Mezigdomide builds on these observations, showing even stronger activity, including in models with markedly reduced CRBN expression or impaired cereblon signaling. Through optimized structural design, it achieves high‐affinity CRBN binding and efficient neosubstrate recruitment despite resistance mechanisms that diminish IMiD efficacy. This enhanced potency correlates with rapid induction of apoptosis, pronounced cell cycle arrest, and broad suppression of transcriptional programs essential for MM cell survival [32].

Both CELMoDs exhibit synergy with standard myeloma agents. Iberdomide enhances the cytotoxicity of bortezomib and provides additive immunologic stimulation when combined with daratumumab. Preclinical studies indicate that CELMoDs improve NK‐cell activation and thereby enhance daratumumab‐mediated ADCC. The combination with carfilzomib also produces cooperative antimyeloma activity.

Emerging data highlight important interactions with T‐cell–redirecting therapies. CELMoDs increase T‐cell proliferation and cytokine production, suggesting a capacity to enhance responses to bispecific antibody. Ex vivo studies further reveal that iberdomide and mezigdomide can improve CAR‐T cell expansion during manufacturing and may support persistence after infusion. These findings have encouraged exploratory strategies integrating CELMoDs before or after CAR‐T therapy [33].

In vivo mouse models confirm that CELMoDs reduce tumour burden, prolong survival, and modulate the bone marrow microenvironment in ways that favour immune‐mediated clearance of malignant plasma cells [34].

## 4 Clinical Development of Iberdomide

Iberdomide entered clinical development in heavily pretreated RRMM populations through the phase I/II CC‐220‐MM‐001 (NCT02773030) trial, a large multicenter study conducted across 42 institutions in Europe, Canada, and the United States. The phase I dose‐escalation cohort enrolled 90 patients, with a median age of 65 years and a median of five prior treatment lines.

In the phase I dose‐escalation cohort, 90 patients (median age 65 years, median five prior lines of therapy) received iberdomide in combination with dexamethasone. These patients represent a highly refractory population previously exposed to lenalidomide, pomalidomide, proteasome inhibitors, and CD38‐targeted monoclonal antibodies. In the dose‐escalation phase, iberdomide plus dexamethasone achieved an overall response rate of 32% at a median follow‐up of 5.8 months, including complete, very good partial, and partial responses. This early signal of efficacy was particularly relevant given the degree of refractoriness and the prior IMiD exposure. As expected from a potent cereblon E3 ligase modulator, hematologic toxicity was prominent: neutropenia occurred in nearly half of patients and reached grade > 3 or higher in 42%; while anemia, thrombocytopenia, and infections (~62%) were also frequent. Five deaths occurred during treatment, mostly due to progressive disease, underscoring the clinical frailty of this late‐line population.

The phase II expansion cohort confirmed these findings in an even more heavily pretreated group of 107 triple‐class refractory patients (median age 64; median six prior lines). At a median follow‐up of 7.7 months, the ORR was 26%, with responses observed across sCR, CR, VGPR, and PR categories. Hematologic toxicity remained the dominant adverse event; grade > 3 neutropenia occurred in 45% of patients. Infectious complications—including episodes of sepsis—continued to represent a clinically meaningful safety concern [21, 35].

Beyond the doublet backbone, iberdomide has also been explored in several rationally designed triplet combinations. In the IberDd cohort—iberdomide, daratumumab, and dexamethasone—an overall response rate of 46% was achieved among 43 patients at a median follow‐up of 4.2 months. Neutropenia remained the most common severe toxicity, occurring in two‐thirds of patients, while anemia was also observed. The IberVd regimen (iberdomide, bortezomib, dexamethasone) produced a response rate of 56% in 25 patients at a median follow‐up of 4.86 months, with neutropenia and thrombocytopenia representing the most frequent grade III–IV adverse events. The IberKd cohort (iberdomide, carfilzomib, dexamethasone), although smaller (*N* = 9), demonstrated a response rate of 50% at a median follow‐up of 5 months. Across all triplets, non‐hematologic grade > 3 toxicities were uncommon. Rash, fatigue, or gastrointestinal events were generally mild, suggesting that the enhanced immunomodulatory profile of iberdomide is not accompanied by disproportionate off‐target systemic toxicity [36].

Iberdomide is also being studied in the post‐transplant maintenance setting. The EMN26 phase II trial (NCT04564703) is investigating iberdomide as maintenance therapy after autologous stem‐cell transplantation in newly diagnosed MM. Interim results analyses of the 1.3 mg and 1.0 mg cohorts show encouraging deepening of responses over successive cycles. Patients entering the study with high‐quality responses—predominantly VGPR or better—yet further conversion to stringent complete responses were documented. After six cycles, the 1.3 mg cohort achieved an overall response rate of 45%, with stringent complete responses rising to 51%; the 1.0 mg cohort showed similarly favorable results. Hematologic toxicity, particularly neutropenia, was dose‐dependent but manageable with standard supportive care. Early 6‐month progression‐free survival exceeded 94% in both cohorts, suggesting a potential role for iberdomide in consolidating deep responses after frontline therapy. A longer follow‐up will clarify durability and long‐term safety [37].

Table 1 summarizes the main characteristics of clinical studies on iberdomide in Multiple Myeloma.

**TABLE 1:** Summary of the principal clinical studies evaluating Iberdomide in Multiple Myeloma.

Study/NCT	Phase and design	Population	Regimen	Efficacy	Grade ≥ 3 toxicities
CC‐220‐MM‐001 (Phase I) NCT02773030	Phase I, multicenter dose‐escalation	*N* = 90; median age 65; median 5 prior lines; heavily pretreated RRMM	Iberdomide + dexamethasone	ORR 32% (1% CR, 9% VGPR, 22% PR); FU 5.8 months	Neutropenia 48% (42% ≥ G3); anemia 39%; thrombocytopenia 20%; infections 62%
CC‐220‐MM‐001 (Phase II) NCT02773030	Phase II, multicenter expansion	*N* = 107; median age 64; median 6 prior lines; all triple‐class refractory	Iberdomide + dexamethasone	ORR 26% (1% sCR, 8% VGPR, 18% PR); FU 7.7 months	Neutropenia 60% (45% ≥ G3); anemia 41%; thrombocytopenia 36%; infections 58%; 2% sepsis
IberDd cohort (NCT02773030)	Phase I/II	*N* = 43; heavily pretreated	Iberdomide + daratumumab + dex	ORR 46%; FU 4.2 months	Neutropenia 67%; anemia 21%
IberVd cohort (NCT02773030)	Phase I/II	*N* = 25	Iberdomide + bortezomib + dex	ORR 56%; FU 4.86 months	Neutropenia 28%; thrombocytopenia 24%
IberKd cohort (NCT02773030)	Phase I/II	*N* = 9	Iberdomide + carfilzomib + dex	ORR 50%; FU 5 months	Neutropenia leading AE; few non‐hematologic ≥ G3 AEs
EMN26 post‐ASCT (NCT04564703)	Phase II, maintenance	*N* = 35 (1.3 mg) + *N* = 34 (1.0 mg)	Iberdomide monotherapy	After 6 cycles: sCR 51% (1.3 mg), 47% (1.0 mg); PFS 6‐months: 94%–97%	Neutropenia ≥ G3: 46% (1.3 mg) vs. 21% (1.0 mg); infections 14% vs. 3%

*Note:* The table reports key design characteristics, baseline patient features, regimen composition, efficacy outcomes according to IMWG criteria, and the most frequent grade ≥ 3 adverse events as defined by CTCAE v5.0. Follow‐up values refer to median time at data cut‐off. Trial identifiers (NCT numbers) are provided for reference.

Abbreviations: AE, adverse event; ASCT, autologous stem‐cell transplantation; BCMA, B‐cell maturation antigen; CR, complete response; dex, dexamethasone; FU, follow‐up; IMiD, immunomodulatory drug; NDMM, newly diagnosed multiple myeloma; ORR, overall response rate; PFS, progression‐free survival; PI, proteasome inhibitor; PR, partial response; RRMM, relapsed/refractory multiple myeloma; sCR, stringent complete response; VGPR, very good partial response.

## 5 Clinical Development of Mezigdomide

The development of mezigdomide has generated compelling evidence supporting the capacity of next‐generation cereblon E3 ligase modulators to induce meaningful responses even in profoundly refractory multiple myeloma. The pivotal first‐in‐human study, CC‐92480‐MM‐001 (NCT03374085), evaluated mezigdomide in combination with dexamethasone in a relapsed/refractory population. The phase I component enrolled 77 patients with a median age of 65 years and extensive prior therapy exposure—median six previous lines (with 78%) having undergone autologous stem‐cell transplantation.

At a median follow‐up of 6.3 months, mezigdomide produced an overall response rate of 25%, a noteworthy outcome given the deep refractoriness of the cohort. The potency of mezigdomide was mirrored by an incidence of hematologic toxicity: neutropenia occurred in 81% of patients and reached grade > 3 severity in 71%, while febrile neutropenia appeared in nearly 10%. Anemia, thrombocytopenia, infectious complications, nausea, and fatigue were also frequent, reflecting the profound cereblon‐mediated IKZF1/IKZF3 degradation characteristic of this agent. Nevertheless, toxicity was considered manageable with dose adjustments and supportive care, and the recommended phase II dose was established as 1.0 mg once daily for 21 days of each 28‐day cycle.

The phase II expansion cohort included 101 patients (median age of 67 years), all with triple‐class refractory MM, and included a sizeable proportion previously treated with BCMA‐directed therapy (30%). Notably, 40% presented with plasmacytomas, underscoring the aggressive biology typical of late‐stage disease. At a median follow‐up of 7.5 months, the ORR reached 41%, including complete and stringent complete responses. Responses were observed in patients with plasmacytomas or prior BCMA‐targeted therapy, supporting the capacity of mezigdomide to overcome biological resistance and target difficult‐to‐eradicate clonal populations that frequently evade other therapeutic modalities.

As anticipated, grade III‐IV neutropenia (77%) remained the most prominent adverse event, with febrile neutropenia occurring in 15%. Most patients required G‐CSF support, but overall, the safety profile was considered acceptable for such a heavily pretreated cohort. The median progression‐free survival was 4.4 months, extending to 5.4 months in patients previously exposed to BCMA‐targeted agents, an encouraging signal in a population with very limited therapeutic alternatives [30, 38].

Mezigdomide has also been assessed in combination regimens in the CC‐92480‐MM‐002 phase I/II study (NCT03989414). The MeziDd cohort—mezigdomide, daratumumab, and dexamethasone—enrolled 56 patients (median age 67 years; median two prior lines). Although less heavily pretreated than those in the monotherapy study, the majority were refractory to both IMiDs and proteasome inhibitors. The ORR reached 75%, with stringent complete, complete, and deep partial responses. Hematologic toxicity remained common but manageable, with grade III–IV neutropenia in just over half of patients and low rates of febrile neutropenia. Infectious complications were present but occurred at lower frequencies than in monotherapy cohorts.

The MeziEd cohort (mezigdomide, elotuzumab, dexamethasone) enrolled 20 patients and achieved an ORR of 45%. Hematologic toxicities followed a similar pattern, with high‐grade neutropenia in 40% and manageable rates of febrile neutropenia and infection.

Collectively, these findings demonstrate that mezigdomide has significant clinical activity both as a doublet with dexamethasone in deeply refractory MM and in combination with monoclonal antibodies in less heavily pretreated settings. Importantly, its activity in individuals previously exposed to BCMA‐directed therapies represents a meaningful advance, given the growing prevalence of immunotherapy resistance. The primary limitation across studies remains the high incidence of severe neutropenia, emphasizing the need for optimized dosing strategies, proactive supportive care, and infection prophylaxis [39].

Table 2 summarizes the main characteristics of the studies on Mezigdomide in Multiple Myeloma.

**TABLE 2:** Summary of the principal clinical studies evaluating Mezigdomide in Multiple Myeloma.

Study/NCT	Phase and design	Population	Regimen	Efficacy	Key grade ≥ 3 toxicities
CC‐92480‐MM‐001 (Phase I) NCT03374085	Phase I, multicenter	*N* = 77; median age 65; median 6 prior lines; 78% prior ASCT	Mezigdomide + dexamethasone	ORR 25% (1% CR, 12% VGPR, 12% PR); FU 6.3 months	Neutropenia 81% (71% ≥ G3); febrile neutropenia 9%; infections 74%
CC‐92480‐MM‐001 (Phase II) NCT03374085	Phase II expansion	*N* = 101; triple‐class refractory; 30% prior anti‐BCMA; 40% plasmacytomas	Mezigdomide + dex	ORR 41% (2% sCR, 3% CR, 20% VGPR); ORR 30% plasmacytoma; 50% post‐BCMA; PFS 4.4 months	Neutropenia 77% (76% ≥ G3); febrile neutropenia 15%; infections 65%
MeziDd cohort NCT03989414	Phase I/II	*N* = 56; median age 67; median 2 prior lines; IMiD‐refractory 83%	Mezigdomide + daratumumab + dex	ORR 75% (4% sCR, 14% CR, 29% VGPR, 29% PR)	Overall ≥ G3 AEs 77%; neutropenia 54%; anemia 11%; thrombocytopenia 7%; infections 20%
MeziEd cohort NCT03989414	Phase I/II	*N* = 20; RRMM, less heavily pretreated	Mezigdomide + elotuzumab + dex	ORR 45% (5% sCR, 5% VGPR, 35% PR)	Neutropenia ≥ G3: 40%; febrile neutropenia 5%; anemia 20%; infections 35%
MeziKd cohortNCT03989414	Phase I/II	*N* = 27; RRMM, less heavily pretreated,1–4 prior lines	Mezigdomide + carfilzomib + dex	ORR 85%; median PFS 13.5 months; median DoR 11.9 months	Neutropenia ≥ G3: 66%; thrombocytopenia 6%; anemia 12%
MeziVd cohort NCT03989414	Phase I/II	*N* = 35 RRMM; 1–3 prior lines	Mezigdomide + bortezomib + dex	ORR ~86%; median PFS ~17.5 months; median DoR ~19.4 months	Neutropenia ≥ G3: 60%; thrombocytopenia 7%; anemia 10%

*Note:* The table reports key design characteristics, baseline patient features, regimen composition, efficacy outcomes according to IMWG criteria, and the most frequent grade ≥ 3 adverse events as defined by CTCAE v5.0. Follow‐up values refer to median time at data cut‐off. Trial identifiers (NCT numbers) are provided for reference.

Abbreviations: AE, adverse event; ASCT, autologous stem‐cell transplantation; BCMA, B‐cell maturation antigen; CR, complete response; dex, dexamethasone; DoR, duration of response; FU, follow‐up; IMiD, immunomodulatory drug; NDMM, newly diagnosed multiple myeloma; ORR, overall response rate; PFS, progression‐free survival; PI, proteasome inhibitor; PR, partial response; RRMM, relapsed/refractory multiple myeloma; sCR, stringent complete response; VGPR, very good partial response.

## 6 CELMoDs Versus IMiDs

CELMoDs represent a structural evolution of IMiDs, specifically designed to overcome cereblon‐mediated resistance observed with lenalidomide and pomalidomide. The enhanced cereblon affinity of iberdomide and mezigdomide facilitates more efficient formation of the CRBN–CUL4A–RBX1–DDB1 complex in its active configuration. As a result, target substrate degradation is deeper and more sustained, resulting in more robust inhibition of IRF4 and MYC and more extensive immunologic activation [40].

These pharmacologic properties translate into clinically meaningful differences. Whereas lenalidomide and pomalidomide frequently lose efficacy in early relapses—post‐lenalidomide maintenance—CELMoDs retain activity despite prior IMiD exposure and refractoriness. This distinction is particularly relevant in the current therapeutic landscape, where patients commonly receive lenalidomide during induction, consolidation, and prolonged maintenance [41].

Regarding toxicity, CELMoDs and IMiDs share broadly similar profiles, with hematologic suppression being the predominant adverse effect. CELMoDs generally induce more pronounced neutropenia, reflecting their higher potency. Importantly, the increased incidence and severity of cytopenias associated with CELMoDs—particularly neutropenia—should be regarded as an on‐target consequence of their enhanced biological activity rather than an off‐target toxicity. Hematopoietic progenitor cells rely on tightly regulated transcriptional programs involving IKZF family members and related pathways. More potent and sustained cereblon‐mediated substrate degradation can therefore disrupt normal hematopoiesis, resulting in dose‐limiting cytopenias. This mechanistic linkage underscores the narrow therapeutic window of CELMoDs and highlights the need for careful dose optimization, intermittent scheduling, and proactive supportive care. Taken together, the superior efficacy and increased hematologic toxicity of CELMoDs represent two sides of the same biological coin, both driven by intensified cereblon engagement and amplified downstream effects [42].

Effective management of neutropenia is therefore essential to maintain treatment intensity while minimizing infectious complications. In clinical trials, neutropenia has been commonly managed through a combination of dose interruptions, dose reductions, and supportive care with granulocyte colony‐stimulating factor (G‐CSF), enabling most patients to resume therapy without permanent discontinuation. Reactive use of short‐acting G‐CSF is appropriate for the management of grade ≥ 3 neutropenia or febrile neutropenia, especially in heavily pretreated or clinically vulnerable patients [36, 39].

Prophylactic strategies may be considered in selected settings, such as patients with baseline cytopenias, extensive prior therapy, or recurrent neutropenia during early treatment cycles. In this context, the use of pegylated G‐CSF (peg‐G‐CSF) may offer practical advantages by providing sustained neutrophil support with reduced injection frequency, particularly for patients treated with intermittent dosing schedules. Although prospective data directly comparing prophylactic and reactive growth factor strategies in CELMoD‐treated patients are limited, available clinical trial experience suggests that early and proactive hematologic support can reduce treatment interruptions and improve overall tolerability [43].

The broader immunologic effects of CELMoDs extend their utility beyond the antimyeloma effects observed with IMiDs alone. The capacity to potentiate ADCC, improve T‐cell activation, and enhance NK‐cell function positions CELMoDs as highly valuable partners for combination therapy with monoclonal antibodies and T‐cell–redirecting agents [44].

Compared with classical IMiDs, CELMoDs were rationally designed to overcome key mechanisms of resistance to lenalidomide and pomalidomide, most notably impaired cereblon (CRBN) function. Reduced CRBN expression or suboptimal assembly of the CRL4^CRBN E3 ubiquitin ligase complex can limit IMiD efficacy by preventing efficient ubiquitination and degradation of critical neosubstrates, including IKZF1 and IKZF3. Iberdomide and mezigdomide exhibit substantially higher CRBN‐binding affinity, resulting in more stable and productive E3 ligase complex formation and deeper, more sustained degradation of Ikaros and Aiolos, even in the context of partial CRBN deficiency. This enhanced target engagement leads to more pronounced suppression of the IRF4–MYC transcriptional axis, collapse of myeloma‐specific survival programs, and restoration of antitumor activity in IMiD‐refractory disease, while concurrently amplifying T‐ and NK‐cell–mediated immune effects [33].

Despite this increased potency, resistance to CELMoDs can still emerge. Similar to IMiDs, CRBN remains a central resistance mechanism, although more profound disruptions appear to be required to fully abrogate CELMoD activity. Additional resistance pathways likely include adaptive transcriptional reprogramming downstream of IKZF1/3 loss, activation of alternative survival signaling pathways that bypass IRF4–MYC dependence, and microenvironmental or immune escape mechanisms that attenuate immunomodulatory effects. Further elucidation of these processes will be essential for optimizing treatment sequencing, rational combination strategies, and patient selection as CELMoDs are increasingly integrated into clinical practice [44].

Table 3 summarizes key pharmacologic and clinical differences between CELMoDs and IMiDs.

**TABLE 3:** Comparative overview of classical IMiDs and next‐generation CELMoDs, summarizing structural and mechanistic differences, cereblon binding characteristics, transcriptional impact, immunomodulatory activity, clinical development stage, and toxicity patterns relevant to the treatment of Multiple Myeloma.

Feature	IMiDs (Thalidomide, Lenalidomide, Pomalidomide)	CELMoDs (Iberdomide, Mezigdomide)
Drug class	Classical immunomodulatory drugs (IMiDs)	Next‐generation cereblon E3 ligase modulators (CELMoDs)
Cereblon binding affinity	Moderate affinity; limited stabilization of CRBN complex	Markedly increased affinity; superior stabilization of CRBN in the “closed active” conformation
CRBN conformational effect	Partial allosteric modulation	Enhanced conformational locking → improved neosubstrate presentation and degradation
IKZF1/IKZF3 degradation	Effective but slower and less complete	Rapid, deep, and sustained degradation of IKZF1 and IKZF3; stronger IRF4 and MYC suppression
Activity in IMiD‐refractory disease	Often reduced or lost, particularly after lenalidomide maintenance	Retained even with low CRBN expression; active in lenalidomide and pomalidomide‐refractory myeloma
Immunomodulatory potency	T‐cell co‐stimulation, NK‐cell activation, enhanced immune synapse formation	Greater T‐cell proliferation, enhanced cytokine release, stronger NK‐cell cytotoxicity and ADCC
Synergy with monoclonal antibodies	Good synergy (especially lenalidomide + anti‐CD38)	Potent synergy with daratumumab, elotuzumab, bispecific antibodies, and BCMA‐directed immunotherapies
Clinical stage	Approved in frontline, maintenance, and RRMM	Phase II–III development; promising in early relapse (iberdomide) and in triple/penta‐refractory MM (mezigdomide)
Typical dosing schedule	Oral daily days 1–21 of 28‐day cycles	Oral daily days 1–21 of 28‐day cycles; low‐mg dosing (≈1.0–1.6 mg) due to higher pharmacodynamic potency
Dominant toxicities	Cytopenias, fatigue, rash, GI toxicity, infections	More pronounced neutropenia (especially with mezigdomide); infectious complications require prophylaxis and G‐CSF use
Ideal therapeutic setting	NDMM, post‐ASCT maintenance, early RRMM	Iberdomide: early relapse and post‐ASCT maintenance; Mezigdomide: triple−/penta‐refractory MM, post‐BCMA failure

Abbreviations: ADCC, Antibody‐Dependent Cell‐Mediated Cytotoxicity; ASCT, autologous stem‐cell transplantation; BCMA, B‐cell maturation antigen; CRBN, cereblon; G‐CSF, Granulocyte‐Colony Stimulating Factor; NDMM, newly diagnosed multiple myeloma; NK, Natural Killer; RRMM, relapsed/refractory multiple myeloma.

## 7 Future Directions

The structural and mechanistic attributes of iberdomide and mezigdomide make them attractive candidates for a wide array of therapeutic combinations. Enhanced NK‐cell activation observed with CELMoDs provides a compelling rationale for combining them with CD38 monoclonal antibodies such as daratumumab or isatuximab. The EXCALIBER‐RRMM trial exemplifies this approach by evaluating iberdomide in combination with daratumumab and dexamethasone in early‐relapsed MM [37].

The rationale for combination with proteasome inhibitors is equally compelling. Proteasome inhibitors disrupt protein homeostasis and induce apoptosis in MM cells, while CELMoDs weaken survival pathways through transcription factor degradation. Preclinical models consistently demonstrate synergy, and ongoing clinical trials will determine whether this translates into improved outcomes.

Integration with T‐cell–redirecting therapies represents one of the most promising avenues for CELMoD development. Bispecific antibodies targeting BCMA or GPRC5D rely on T‐cell activation and proliferation. CELMoDs enhance these processes, potentially producing deeper and more durable responses. This combination may also mitigate T‐cell exhaustion and support persistence during therapy [45].

CELMoDs also play a role in CAR‐T cell therapy. Both iberdomide and mezigdomide enhance T‐cell expansion ex vivo, suggesting that their incorporation into CAR‐T cell manufacturing could yield more potent cell products. Additionally, their immune‐stimulatory effects post CAR‐T infusion may sustain T‐cell persistence and contribute to long‐term disease control.

In the post‐ASCT maintenance setting, CELMoDs may offer advantages over lenalidomide, providing deeper immunomodulation and enhanced minimal residual disease (MRD) clearance. Ongoing studies are evaluating this potential [46].

Despite promising mechanistic and early clinical data, several questions remain. Long‐term toxicities—particularly cumulative cytopenias and infection risk—require further characterization. The mechanisms of acquired resistance to CELMoDs have not been fully understood, and it remains unclear whether prolonged exposure will select for CRBN‐independent survival pathways analogous to those observed with long‐term IMiD therapy.

The absence of mature Phase III results limits the precise placement of CELMoDs within the MM treatment algorithm. Optimal sequencing relative to CAR‐T cells, bispecific antibodies, and proteasome inhibitors remains to be defined. Data on newly diagnosed MM are also limited, though ongoing trials clarify potential roles in induction or maintenance [47].

The impact of CELMoDs on T‐cell exhaustion in combination with immunotherapies remains incompletely understood. Finally, the heterogeneity of MM and its dynamic evolution under therapeutic pressure highlight the need for long‐term clinical and translational investigation [48].

## 8 Conclusions

Iberdomide and mezigdomide represent a significant advance in cereblon‐targeting therapeutics for multiple myeloma. By enhancing substrate degradation, increasing cereblon affinity, and exerting a potent immunomodulatory effect, CELMoDs effectively overcome many resistance mechanisms that limit IMiD efficacy.

Early clinical responses observed in heavily pretreated RRMM—including triple‐ and penta‐refractory populations—highlight their therapeutic potential. Iberdomide appears particularly suitable for early‐relapse settings and for integration into combination regimens with monoclonal antibodies or proteasome inhibitors. Mezigdomide, with its more robust pharmacodynamic profile, may address an urgent need in deeply refractory disease.

**FIGURE 3:**
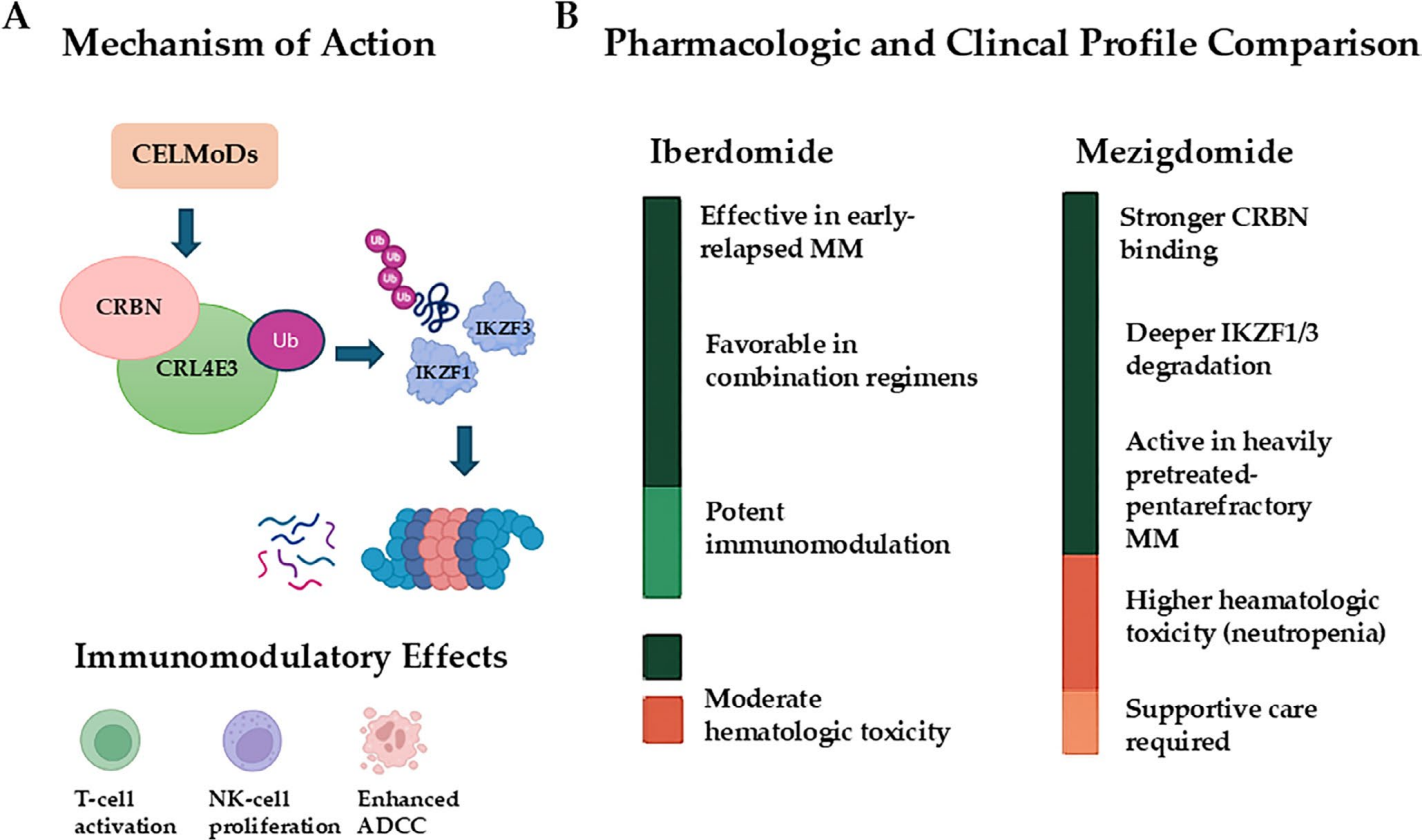
Mechanistic and Clinical Comparison of Iberdomide and Mezigdomide in Multiple Myeloma. Panel (A) illustrates the molecular mechanism of action of CELMoDs. Both iberdomide and mezigdomide bind cereblon (CRBN) within the CRL4 E3 ubiquitin ligase complex, promoting recruitment, ubiquitination, and proteasomal degradation of transcription factors IKZF1 and IKZF3. This leads to direct antimyeloma effects and enhanced immunomodulatory activity through increased T‐ and NK‐cell function. Panel (B) summarizes key clinical distinctions. Iberdomide provides flexibility for combination therapy and is particularly effective in early‐relapse multiple myeloma, whereas mezigdomide exhibits a more robust pharmacodynamic profile with activity in heavily pretreated, penta‐refractory patients. However, mezigdomide's increased potency is associated with higher rates of hematologic toxicity, primarily neutropenia, requiring careful monitoring and supportive care. These complementary profiles suggest that clinical selection between the two agents should consider disease stage, prior therapy exposure, and tolerance to hematologic adverse events.

The complementary profiles of iberdomide and mezigdomide (Figure 3)—where iberdomide provides flexibility for combination therapy in early‐relapse MM and mezigdomide delivers potent activity in heavily pretreated, penta‐refractory populations—suggest that drug selection may be guided by disease stage, prior therapy exposure, and patient tolerance to hematologic adverse events. Mezigdomide's increased potency, however, is associated with higher rates of hematologic toxicity, primarily neutropenia, emphasizing the need for careful monitoring and supportive care.

As the MM treatment landscape increasingly emphasizes immunotherapy‐based approaches, CELMoDs offer a unique opportunity to strengthen immune effector function, enhance cytotoxicity, and synergize with CAR‐T and bispecific therapies. Ongoing Phase III trials will be crucial in defining their ultimate therapeutic positioning.

Overall, CELMoDs hold the potential of reshaping the MM treatment continuum, deepening responses, extending remission durations, and overcoming resistance barriers inherent to traditional IMiDs.

## Author Contributions

Enrica Antonia Martino, Santino Caserta, Mamdouh Skafi, Fortunato Morabito, Massimo Gentile: Conceptualization. Enrica Antonia Martino, Francesco Mendicino, Ernesto Vigna, Antonella Bruzzese, and Fortunato Morabito: Methodology. Enrica Antonia Martino, Santino Caserta, Fortunato Morabito, Massimo Gentile Writing – Original Draft Preparation. Enrica Antonia Martino, Santino Caserta, Mamdouh Skafi, Fortunato Morabito, Massimo Gentile: Writing, review, and editing. All authors have read and agreed to the published version of the manuscript.

## Funding

The authors have nothing to report.

## Ethics Statement

The authors have nothing to report.

## Conflicts of Interest

The authors declare no conflicts of interest.

## Data Availability

Data sharing does not apply to this article, as no new data were generated or analyzed in this study.
